# Tracheal chondrosarcoma: A case report, systematic review, and pooled analysis

**DOI:** 10.1002/cnr2.1537

**Published:** 2021-09-02

**Authors:** Mitchell Heuermann, Simon Bekker, Thomas Czeczok, Stacie Gregory, Arun Sharma

**Affiliations:** ^1^ Department of Otolaryngology—Head and Neck Surgery Southern Illinois University School of Medicine Springfield Illinois USA; ^2^ Department of Radiology Southern Illinois University Springfield Illinois USA; ^3^ Associated Pathologists Springfield Illinois USA

**Keywords:** chondrosarcoma, head and neck sarcoma, systematic review, tracheal cancer, tracheal chondrosarcoma

## Abstract

**Background:**

Tracheal chondrosarcoma is a rare malignancy, and formal treatment guidelines have not been established due to the lack of high quality studies. Best evidence at this time is limited to case reports.

**Aim:**

Explore the role of surgical intervention, radiation therapy, and chemotherapy, and the long‐term outcomes for these interventions for tracheal chondrosarcoma.

**Methods and Results:**

A literature search was performed using PubMed (1959–2020) and ResearchGate (1959–2020) using medical subject heading terms “tracheal chondrosarcoma” OR “trachea chondrosarcoma.” Additional reports were identified within reviewed articles and included for review. Articles pertaining to chondrosarcomas of the lung, bronchus, larynx, or other head and neck subsites were excluded. Cases of chondromas were excluded. Thirty‐five patients with tracheal chondrosarcoma were identified in the literature since 1959. Advanced age was significantly associated with recurrent or persistent disease (*p* = .003). The majority (77%) of cases were treated with open surgical resection, with an open approach and negative surgical margins being significantly associated with being disease‐free after treatment (*p* = .001 and *p* < .001, respectively). Adjuvant radiotherapy was reserved for those unfit for surgery or for recurrent disease. Tumor size, extra‐tracheal extension, tumor calcification, location, and initial diagnosis were not associated with tumor recurrence.

**Conclusion:**

Non‐metastatic tracheal chondrosarcoma can be treated by adequate surgical resection, with little to no role for adjuvant radiotherapy or chemotherapy. Open surgery and negative margins were associated with oncologic control, while advanced age was associated with recurrent or persistent disease.

## 1 INTRODUCTION

Chondrosarcomas are a rare malignancy, comprising only 0.1% of all head and neck cancer. A majority of cases arise from the bony tissue of the face and paranasal sinus cavities, but less commonly arise from the cartilaginous tissues of the larynx or other soft tissue.^1^ While tumorigenesis is incompletely understood, head and neck chondrosarcomas have been proposed to arise from malignant degeneration of cartilage remnants, mesenchymal pluripotent cells, ossified cartilage, and from isolated endochondromas.^2^ Several syndromes have also been associated with a higher incidence of chondrosarcoma, including Ollier disease, Maffucci syndrome, and Paget's disease of the bone.^2^

Within the head and neck, chondrosarcoma of the trachea accounts for an even smaller subset of all chondrosarcomas, with only 34 cases reported in the literature between 1959 and 2020. Because of this, evidence for treatment is limited to these case reports, although surgery has historically been the mainstay of treatment.

To determine appropriate treatment of tracheal chondrosarcomas, we reviewed case reports and case series in the literature that reported on surgical and nonsurgical treatment and outcomes. In this review, we discuss a case of proximal tracheal chondrosarcoma treated at our institution, and report treatment and outcome results of a systematic review and pooled analysis of the available clinical literature. The goal was to assess characteristics of patients with tracheal chondrosarcoma, treatment, and oncologic outcomes.

## 2 MATERIALS AND METHODS

**FIGURE 1:**
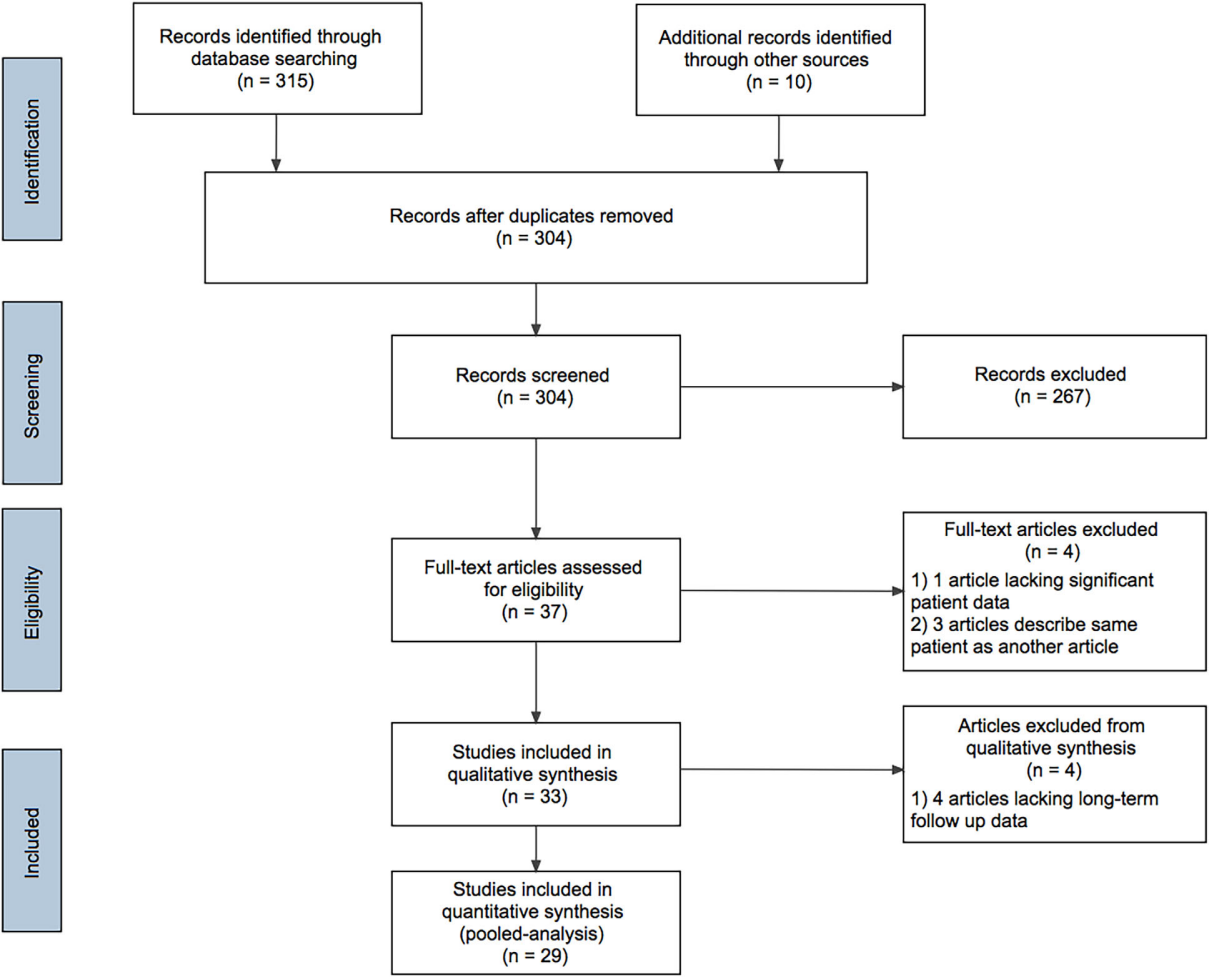
A PRISMA diagram of systematic literature review

A literature search was performed using PubMed (1959–2020) and ResearchGate (1959–2020) using medical subject heading terms “tracheal chondrosarcoma” and “trachea chondrosarcoma”; these databases were searched on May 13, 2000. Further case reports were identified within reviewed articles and included for review. Articles containing cases of chondrosarcomas primary to the lung, bronchus, larynx, or other head and neck subsites were excluded. Articles with cases of chondromas or those lacking the majority of patient data were also excluded. Articles in non‐English languages were included for review and translated using Google Translate's (Alphabet Inc, Menlo Park, CA) whole document translator. Data was independently extracted from the reports by the first author, and any ambiguous data not explicitly stated was recorded based on the consensus of the first and corresponding authors. Additional data missing from the reports that could be verified through the corresponding author was included. Case reports describing the same patient were consolidated. The details of the literature search and review are presented as a PRISMA diagram in Figure 1. Variables abstracted from individual studies were patient age, sex, presenting symptom(s), duration of symptoms, initial diagnosis, tracheal site, tumor grade, tumor size, presence of extratracheal extension, presence of calcifications, treatment modality or modalities, presence of gross residual disease, tumor recurrence, and follow‐up duration.

Data from the case reports were pooled. Continuous variables were compared with the Wilcoxon ranksum test. Categorical variables were compared with chi‐squared test and, when appropriate, Fisher's exact test. *p* < .05 was set at the cutoff for statistical significance. All statistical analysis was performed with Stata/SE 14.2 (College Station, TX).

## 3 CASE DESCRIPTION

A 66‐year‐old male with a history of hypertension, type 2 diabetes mellitus, hyperlipidemia, aortic aneurysm, and a remote history of colon cancer presented to the emergency department of our tertiary care academic medical center with an acute onset of hemoptysis and dyspnea. He had reported mildly increased dyspnea with exertion over the past 6 months.

**FIGURE 2:**
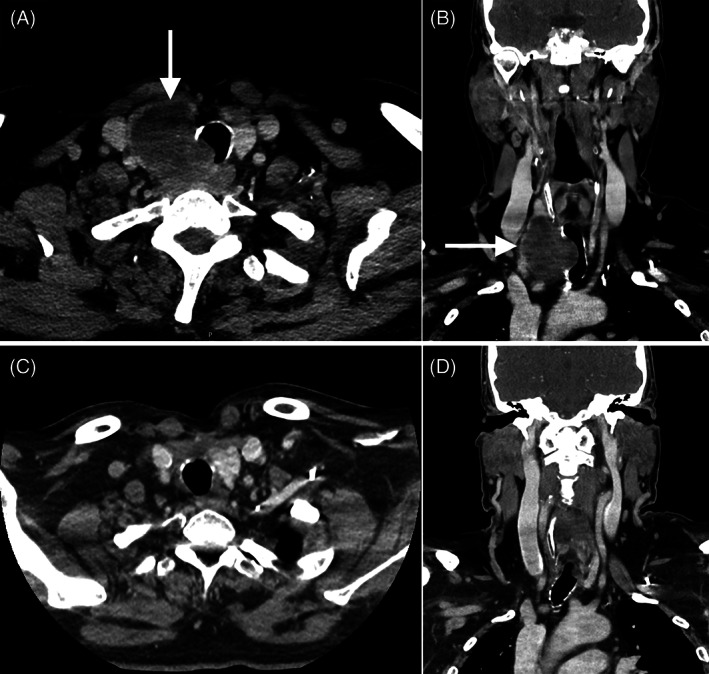
Contrast enhanced axial and coronal computed tomography imaging (A) and (B) on presentation and (C) and (D) at 3 months post‐operative

Neck computed tomography imaging with contrast showed >75% intraluminal tracheal narrowing secondary to a calcified 4.8 × 3.6 × 5.6 cm mass involving right thyroid lobe and proximal trachea, splaying rings 1 and 2, but sparing cricoid (Figure 2A,B). An 11 mm paratracheal node (level VI) was also noted. Ultrasound‐guided core biopsy of the mass showed low‐grade chondrosarcoma. Pre‐operative computed tomography imaging with contrast of the chest, abdomen, and pelvis showed no evidence of distant metastasis.

**FIGURE 3:**
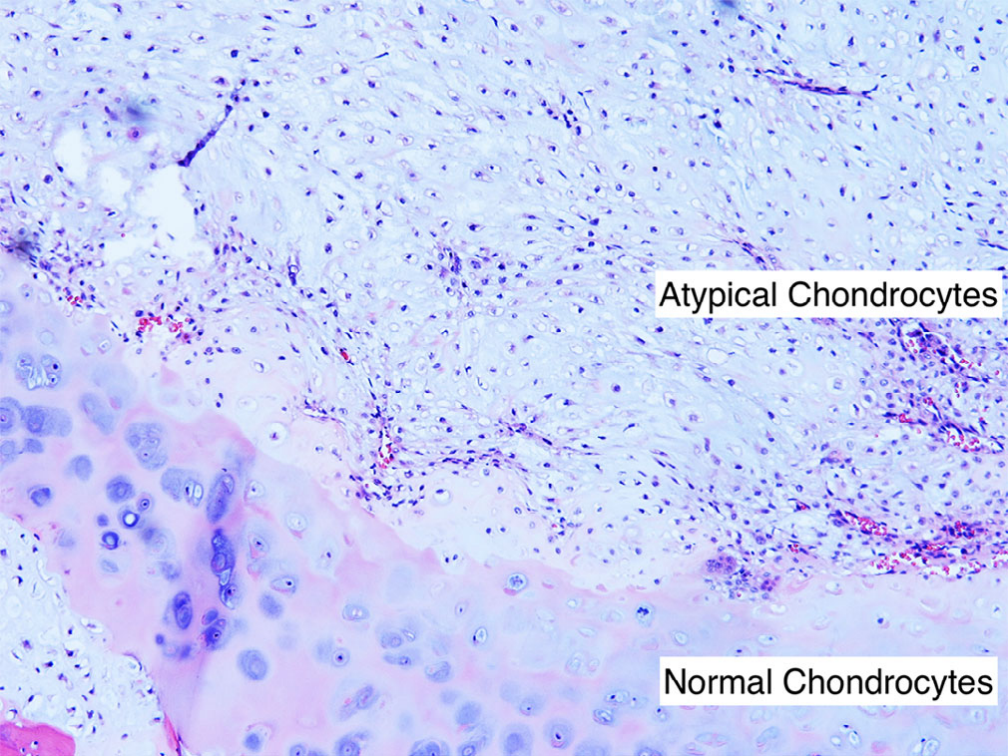
Histologic section of the tumor. The upper right shows a hypercellular cartilaginous process as compared to the adjacent benign cartilage, seen in the bottom of the image. Neoplastic chondrocytes show mild atypia

The patient then underwent definitive treatment with an en‐bloc right thyroid lobectomy and proximal tracheal resection, right central neck dissection levels 6 and 7, and primary tracheal anastomosis. A tension‐free end‐to‐end tracheal anastomosis was obtained and no Grillo stitch was placed. He was extubated uneventfully on postoperative day 1. His post‐operative course was complicated by hospital‐acquired pneumonia, which resolved with antibiotics. He was downgraded from the intensive care unit on postoperative day 6, and discharged to home on day 7. Final pathology (Figure 3) was consistent with a pT3N0M0 (American Joint Committee on Cancer [AJCC] 8th edition staging), grade 2 tracheal chondrosarcoma with negative margins. No adjuvant therapy was given. Repeat bronchoscopies at 1 and 4 months from the initial tracheal resection and CT imaging 3 months from surgery (Figure 2C,D) showed no evidence of residual disease or stenosis. He has remained disease free at 16 months post‐treatment.

## 4 RESULTS

Our literature search yielded 32 English language publications describing a total of 33 patient reports^3^, ^4^, ^5^, ^6^, ^7^, ^8^, ^9^, ^10^, ^11^, ^12^, ^13^, ^14^, ^15^, ^16^, ^17^, ^18^, ^19^, ^20^, ^21^, ^22^, ^23^, ^24^, ^25^, ^26^, ^27^, ^28^, ^29^, ^30^, ^31^, ^32^, ^33^ and 5 non‐English language publications reports^34^, ^35^, ^36^, ^37^, ^38^ describing a total of 5 patients. One case report lacked significant patient data and was excluded. Three pairs of cases described the same case and were consolidated.^9^, ^16^, ^17^, ^22^, ^23^, ^37^ Thus, including our own reported case, a total of 35 unique cases were included for analysis. Four patient reports lacked long‐term follow up data, and were also excluded specifically from survival analysis.^11^, ^16^, ^17^, ^19^, ^21^ All studies were case reports (level 5 evidence).

The demographic and tumor characteristics of these are detailed in Tables 1 and S1. The median age at presentation was 68 years (inter‐quartile range [IQR], 58–75 years), and 91% of patients were male. The most common presenting symptom was dyspnea (80%), and 45% of patients were initially misdiagnosed, most commonly with asthma. The distribution among the proximal, middle, and distal trachea was 54%, 17%, and 29%, respectively. Calcifications were seen in 71% of tumors and 74% showed extratracheal extension on pre‐operative imaging (when available). The median tumor size was 3.0 cm (IQR, 2.5–4.0 cm).

**TABLE 1:** Univariate analysis of patient, tumor, and treatment characteristics comparing patients with no recurrent or persistent disease after surgical treatment to those with residual or recurrent disease

Characteristic	All patients (*n* = 35)	Disease‐free after treatment (*n* = 23)	Recurrent or persistent disease after treatment (*n* = 8)	*p* value
Age, median (IQR)	68 (58–75)	64 (54–72)	76.5 (73.5–87)	.003
Sex, male	32 (91%)	21 (91%)	7 (88%)	1.000
Symptoms				
Cough	14 (40%)	7 (30%)	5 (63%)	.206
Dysphagia	2 (6%)	1 (4%)	1 (13%)	.456
Dysphonia	7 (20%)	4 (17%)	2 (25%)	.634
Dyspnea	28 (80%)	20 (87%)	5 (63%)	.161
Hemoptysis	6 (17%)	5 (22%)	1 (13%)	1.000
Odynophagia	1 (3%)	1 (4%)	0	1.000
Pneumonia	2 (6%)	1 (4%)	1 (13%)	.456
Stridor	11 (31%)	7 (30%)	1 (13%)	.642
Wheeze	13 (37%)	8 (35%)	3 (38%)	1.000
Total number of symptoms at presentation, median (IQR)	2 (2–3)	2 (2–3)	2 (1–3.5)	.800
Symptom duration, months, median (IQR)	7.5 (2.5–24)	6 (1–24)	12 (3–12)	.670
Initial diagnosis				1.000
Tracheal mass	18 (51%)	11 (48%)	6 (75%)	
Asthma	7 (20%)	4 (17%)	1 (13%)	
Thyroid mass/cancer	3 (9%)	2 (9%)	0	
COPD	3 (9%)	2 (9%)	1 (13%)	
Angina	1 (3%)	1 (4%)	0	
Pneumonia	1 (3%)	1 (4%)	0	
Unknown	2 (6%)	2 (9%)	0	
Tumor size, cm, median (IQR)	3.0 (2.5–4.0)	3 (2.5–4.0)	3 (2.3–4.0)	.717
Site of disease in trachea				.367
Proximal	19 (54%)	13 (57%)	3 (38%)	
Mid	6 (17%)	3 (13%)	3 (38%)	
Distal	10 (29%)	7 (30%)	2 (25%)	
Tumor grade				.148
1	19 (54%)	14 (61%)	3 (38%)	
2	11 (31%)	6 (26%)	4 (50%)	
3	1 (3%)	0	1 (13%)	
Unknown	4 (11%)	3 (13%)	0	
ETE present				.304
Yes	26 (74%)	18 (78%)	5 (63%)	
No	6 (17%0	4 (17%)	1 (13%)	
Unknown	3 (9%)	1 (4%)	2 (25%)	
Calcifications present				.216
Yes	25 (71%)	18 (78%)	4 (50%)	
No	3 (9%)	2 (9%)	1 (12%)	
Unknown	7 (20%)	3 (13%)	3 (38%)	
Surgical approach				.001
Open	27 (77%)	21 (91%)	2 (25%)	
Endoscopic	8 (23%)	2 (9%)	6 (75%)	
Gross residual disease at time of resection	7 (20%)	1 (4%)	6 (75%)	<.001
Adjuvant RT	3 (9%)	1 (4%)	2 (25%)	.156
Follow‐up time, months, median (IQR)	30 (12–42)	27 (12–48)	36 (12–36)	.837

Abbreviations: IQR, Interquartile range; RT, radiotherapy.

Treatment and outcome data are reported in Tables 1 and S1. Open surgical resection to negative margins was the primary treatment modality in 77% of cases, and of these, only two developed recurrence. Median follow up time was 30 months (IQR, 12–42 months). Endoscopic debulking with or without adjuvant radiotherapy was usually reserved for patients who either refused surgery or were unfit surgical candidates, although this treatment generally resulted in a stable outcome for the patient in spite of residual disease. Chemotherapy was not given in any cases.

**FIGURE 4:**
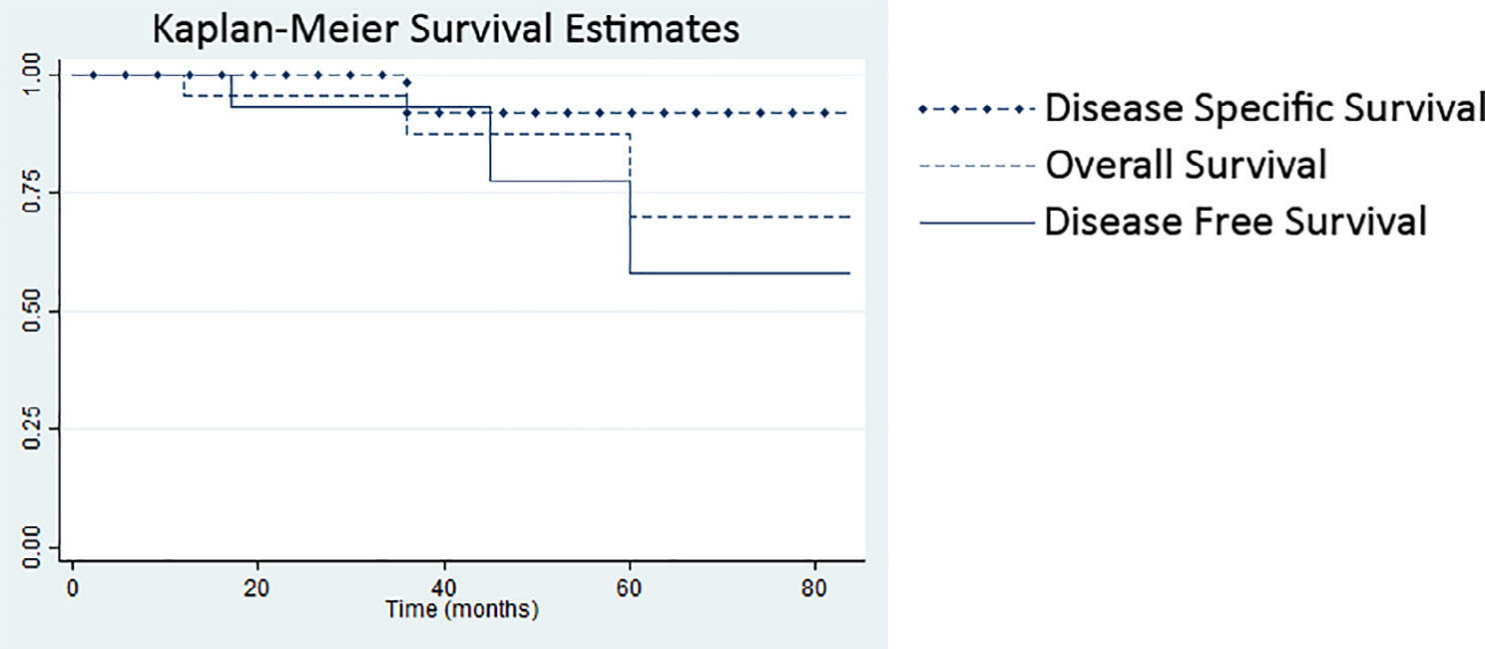
Kaplan–Meier curves for disease free survival, overall survival, and disease specific survival

For patients in whom long‐term follow‐up and outcomes were reported, univariate analysis was performed, comparing the group that developed residual or recurrent disease after definitive treatment to the group that did not develop recurrence (Table 1). Kaplan–Meier curves were generated for overall survival, disease specific survival, and disease free survival and reported in Figure 4. Patients who did not develop recurrence or residual disease were significantly younger than those who did (64 years [IQR 54–72 years] vs. 76.5 years [IQR 73.5–87 years], *p* = .003); sex distribution did not significantly differ between groups. None of the presenting factors, including symptoms, total number of presenting symptoms, symptom duration, or initial diagnosis, differed significantly between groups. Similarly, the tumor characteristics of size, trachea subsite, presence of extratracheal extension, or presence of calcifications did not differ significantly between groups. Tumor grade approached significance (*p* = .148), with the percentage of higher grade tumors being higher in the group with recurrent or residual disease. The group developing recurrence or residual disease was significantly more likely to have had an endoscopic resection or gross residual disease after definitive surgical treatment (*p* = .001 and *p* < .0001). The use of adjuvant radiotherapy was more common in the group developing residual or recurrent disease, but this did not reach significance (*p* = .156).

## 5 DISCUSSION

Chondrosarcomas within the head and neck are rare, and represent only 0.1% of all tumors.^1^ The subset of tracheal chondrosarcomas comprises an even rarer subset, with only 34 cases being reported in the past 60 years. Given the rarity of tracheal chondrosarcoma, there is a paucity of specific guidelines for treatment. Thus, best evidence for treatment is limited to the results of case reports and extrapolation from outcomes of chondrosarcomas found elsewhere in the body.

The overall median age at diagnosis was 68 years (IQR, 58–75 years), which is greater than the reported median age of diagnosis for all head and neck chondrosarcomas (51.0 years).^1^ Additionally, patients who did not develop recurrence or residual disease were significantly younger than those who did (64 years [IQR 54–72 years] vs. 76.5 years [IQR 73.5–87 years], *p* = .003). It is possible that in this situation, age represents a surrogate marker for overall health status, which may preclude a large surgical operation. Patients developing recurrent or residual disease were also less likely to have been treated with open resection, supporting this consideration. A difference in overall patient health may also explain the discrepancy between disease specific survival and overall survival noted in the Kaplan Meier analysis (Figure 4). The proportion of males affected (91%) exceeds the proportion of males affected for all head and neck chondrosarcomas (54.5%).^1^ The reasons for these demographic discrepancies are unknown, but could be artifactual given the relatively low sample size. Alternatively, they could represent some differences of tumor biology that have yet to be elucidated.

Tracheal chondrosarcomas tend to be slow growing and the onset of symptoms insidious. The median duration of symptoms prior to presentation was 7.5 months (IQR, 2–24 months), with only seven patients presenting with symptoms lasting 1 month or less. Symptoms at presentation most often result from airway obstruction, which typically does not become symptomatic until >75% luminal obstruction occurs.^13^ Dyspnea and cough were the two most common presenting symptoms at 80% and 40%, respectively. Given the nonspecific symptoms and typical lack of external findings, it is unsurprising that many cases are initially misdiagnosed. Although a majority (55%) of patients were correctly diagnosed with a tracheal mass at the time of presentation, 29% were incorrectly diagnosed as asthma or COPD exacerbations, and 9% with thyroid masses. In four cases, the diagnosis of chondrosarcoma was made in the context of a previously known tracheal endochondroma,^9^, ^10^, ^24^, ^33^, ^37^ which is consistent with reports of malignant transformation of chondromas in other parts of the body.^39^ In one case,^9^, ^37^ the diagnosis of low‐grade (grade I) chondrosarcoma was made less than a year out from the resection of the chondroma. Given the subtle differences in grading between chondroma and low‐grade chondrosarcoma,^2^ it is possible that the originally diagnosed chondromas actually represented early stage, low‐grade chondrosarcomas.

As a rule, optimal treatment should consist of surgical excision to negative margins, usually including a segmental tracheal resection with primary end‐to‐end anastomosis. A review of the surgical steps for a tracheal resection has been reported by Mathisen.^40^ In cases where there is tumor extension to the subglottis or other laryngeal structures, total or partial laryngectomy should be considered. Twenty‐one of 25 cases undergoing open resection reported on follow up and long term outcomes. Of these, all but two showed no evidence of disease at their latest reported follow up date (median 30 months, IQR 12–42 months).

Attempted shave resection or endoscopic resection is not recommended, except in cases of temporization or palliation of the airway. Only one patient was reported to have been successfully cleared of his disease endoscopically, though notably this tumor was described as pedunculated.^3^ Endoscopic resection was significantly more common in the group developing residual or recurrent disease (75% vs. 9%, *p* = .001), likely due to incomplete surgical resection. All but two patients developing recurrence or progression of their disease had grossly residual disease after their definitive surgical intervention,^4^, ^13^, ^24^, ^25^, ^30^, ^33^ and the proportion of those with residual disease was significantly greater in this group compared to those not developing recurrence (75% vs. 4%, *p* < .001), highlighting the importance of obtaining adequate oncologic margins.

Adjuvant radiation therapy (RT) has thus far played only a limited role in tracheal chondrosarcoma management, as they are generally considered to be radioresistant. The one patient who underwent 50 Gy of adjuvant conformal RT in spite of reported surgical clearance of his disease ultimately developed local recurrence of his chondrosarcoma ~4 years later, which was successfully treated by re‐resection.^38^ The remaining instances in which RT was given were limited to cases in which surgical resection was incomplete or contraindicated. One of these two patients remained stable with disease for at least 12 months after receiving 40 Gy.^13^ Interestingly, the other patient was successfully cleared of disease with endoscopic debulking followed by 60 Gy of adjuvant external‐beam radiotherapy to the primary site and 40 Gy to the mediastinum, remaining disease free for the duration of his 84 month‐follow up period.^18^ A recent retrospective cohort study of the National Cancer Database identified that 680 of 5427 patients with chondrosarcoma were treated with radiotherapy. Patients undergoing radiotherapy were more likely to have a primary tumor in the head and neck or spine, have high or intermediate grade tumors, have positive surgical margins, or have undergone concurrent chemotherapy compared to those who did not receive adjuvant radiation. Radiation‐related factors demonstrating the best 5‐year survival included high‐dose therapy (>60 Gy) and treatment with advanced radiotherapy options (proton beam therapy, intensity‐modulated RT, stereotactic radiosurgery), particularly among patients with positive surgical margins.^41^

No patients in this systematic review were treated with chemotherapy, but this may be considered for patients with advanced disease. In chondrosarcomas of the bone, chemotherapy has been used in select cases with variable success. In one review of 180 patients with advanced chondrosarcoma treated with chemotherapy, 2 patients had complete response, 22 had partial response, 67 had stable disease, and 72 had progression of their disease. Chemotherapy was most effective at improving progression‐free survival with better patient performance status, fewer metastatic sites, and use of combination agents.^42^

For patients with contraindications to open surgical resection, radiation, and chemotherapy, observation alone after endoscopic debulking is reasonable. Three of four patients who underwent endoscopic debulking without adjuvant treatment remained relatively symptom free for up to 36 months.^4^, ^24^, ^25^, ^33^ Indeed, while disease free survival dropped to 58% at 5 years, disease specific survival remained above 90% (Figure 4). This highlights the usual indolent nature of tracheal chondrosarcoma, particularly for low‐grade tumors.

In almost all cases, tracheal chondrosarcoma has not been shown to metastasize. While local progression with incomplete surgical excision is common, only one case reported recurrence with distant metastatic disease after initial complete surgical excision.^10^ This case was atypical in that it was the only reported case of high grade (grade 3) chondrosarcoma, it was one of several cases thought to represent malignant transformation of a previously‐excised tracheal chondroma, and it was the only case in which the patient was reported to have died of their disease. After resection of the chondrosarcoma, this patient developed bony metastases to the ribs at 17 and 30 months post‐operatively, and ultimately developed widespread metastases, succumbing to his disease 36 months after his initial resection. Notably, this patient never received adjuvant radiotherapy or chemotherapy. These results mirror the behavior of bone chondrosarcomas, in which the rate of metastasis is 0%–10% for grade I tumors, 10%–50% for grade II tumors, and 50%–70% for grade 3 tumors.^43^

Our study has limitations. Because of the rarity of the disease, all included studies were limited to case reports. Many of these, especially earlier‐published reports, lacked important data about tumor characteristics, treatments, or outcomes; more consistent reporting is needed.

## 6 CONCLUSION

As most tracheal chondrosarcomas present as low grade, nonmetastatic tumors, they are treatable with surgical resection alone, which results in long‐term oncologic cure in most cases. Available evidence suggests radiotherapy and chemotherapy are generally not indicated, although they could be considered for advanced disease or in cases where surgery is a contraindication. Given the rarity of the condition, future cases of tracheal chondrosarcoma should be rigorously reported to allow for better characterization of its presentation, appropriate treatment, and outcomes.

## CONFLICT OF INTEREST

The authors have stated explicitly that there are no conflicts of interest in connection with this article.

## ETHICAL STATEMENT

The subject of this case report has given consent to participate and to publish this account. The institutional board review was not required at our institution.

## AUTHOR CONTRIBUTIONS

**M.H.:** Conceptualization; data curation; formal analysis; investigation; methodology; project administration; visualization; writing – original draft; writing – review and editing. **S.B.:** Data curation; investigation; writing – review and editing. **T.C.:** Data curation; investigation; writing – review and editing. **S.G.:** Conceptualization; investigation; methodology; writing – review and editing. **A.S.:** Conceptualization; data curation; formal analysis; investigation; methodology; project administration; supervision; visualization; writing – review and editing.

## Supporting information


**TABLE S1** Demographic and tumor characteristics of 35 patients with tracheal chondrosarcoma. All studies included represent case reports (level 5 evidence). Abbreviations: NR, not reported; M, male; F, female; OR, open resection; ED, endoscopic debulking; ER, endoscopic resection; RT, radiation therapy; RD, residual disease

## Data Availability

The data that support the findings of this study are available from the corresponding author upon reasonable request.
