# Supplementary material for: Tracheal chondrosarcoma: A case report, systematic review, and pooled analysis
Source: Cancer Rep (Hoboken). 2021 Sep 2;5(7):e1537. doi: 10.1002/cnr2.1537 (PMC9327659; doi:10.1002/cnr2.1537)
Supplement: Supplementary file 1 — TABLE S1 Demographic and tumor characteristics of 35 patients with tracheal chondrosarcoma. All studies included represent case reports (level 5 evidence). Abbreviations: NR, not reported; M, male; F, female; OR, open resection; ED, endoscopic debulking; ER, endoscopic resection; RT, radiation therapy; RD, residual disease [file CNR2-5-e1537-s001.docx]

| First author | Year | Age (y) | Sex | Grade | Extra-tracheal Extension | Diameter (cm) | Calcifi-cation | Definitive Treatment | Gross residual disease | Recurrence | Follow up (months) |
| --- | --- | --- | --- | --- | --- | --- | --- | --- | --- | --- | --- |
| Jackson et al^3^ | 1959 | 32 | M | NR | NR | NR | NR | ER | No | No | 72 |
| Daniels et al^4^ | 1967 | 73 | M | 2 | NR | 2.5 | Yes | ED | Yes | RD | 36 |
| Fallahnejad et al^5^ | 1972 | 48 | F | NR | Yes | 4.0 | NR | OR | No | No | 60 |
| Weber et al^6^ | 1978 | 71^¶^ | M^¶^ | NR | Yes | 3.0 ^¶^ | Yes | OR^¶^ | No^¶^ | No^¶^ | 60^¶^ |
| Slasky et al^7^ | 1985 | 58 | M | 2 | Yes | 4.0 | Yes | OR | No | No | 30 |
| Arévalo et al^8^ | 1986 | 74 | M | 1 | Yes | 2.5 | Yes | OR | No | No | 12 |
| Matsuo et al^9^,  Mine et al^37^ ^‡^ | 1987 | 72 | M | 1 | Yes | 4.5 | Yes | OR | No | No | 34 |
| Salminen et al^10^ | 1990 | 68 | M | 3 | Yes | 10 | Yes | OR | No | Local, distant | 36 |
| Kaneda et al^34^ | 1993 | 64 | M | 2 | No | 2.3 | Yes | OR | No | No | 12 |
| Leach et al^11^ | 1994 | 72 | M | NR | Yes | 6.5 | Yes | OR | No | NR | NR |
| Kiriyama et al^12^ | 1997 | 54 | M | 1 | No | 2.0 | Yes | OR^††^ | No | No | 42 |
| Hervas et al^36^ | 1997 | 84 | M | 1 | Yes | 2.0 | Yes | OR | No | No | 36 |
| Farrell et al^13^ | 1998 | 87 | M | 2 | Yes | 3.0 | No | ED, RT^§§^ | Yes | RD | 12 |
| Aznar et al^35^ | 2001 | 49 | M | 2 | Yes | NR | Yes | OR | No | No | 48 |
| Maish et al^14^ | 2003 | 78 | M | 1 | Yes | NR | No | OR^††^ | No | No | 6 |
| Umezu et al^15^ | 2008 | 34 | M | 1 | Yes | 2.5 | Yes | OR | No | No | 76 |
| Wagnetz et al^16^,  de Almeida et al^17^ ^‡^ | 2009 | 34 | M | 2 | No | 2.0 | Yes | OR^††^ | No | NR | NR |
| Mendonça et al^18^ | 2010 | 72 | M | 1 | Yes | 3.0 | Yes | ED, RT^‡‡^ | Yes | No | 84 |
| Mirza et al^19^ | 2010 | 63 | M | 1 | Yes | NR | NR | OR | No | NR | NR |
| Delaere^20^ | 2012 | 64 | M | 1 | Yes | 9 | Yes | OR | No | No | 6 |
| Mohajeri et al^21^ | 2013 | 74 | M | 1 | Yes | 4.0 | Yes | OR | No | NR | NR |
| Scherl, et al^22^,  Darr et al^23^ ^‡^ | 2013 | 80 | M | 2 | Yes | 3.0 | Yes | OR | No | No | 36 |
| Maia et al^24^ | 2016 | 75 | F | 1 | Yes | 3.0 | Yes | ED^‡‡^ | Yes | RD | 9 |
| Ghattas et al^25^ | 2016 | 91 | M | 1 | No | 1.5 | NR | ED | Yes | RD | NR |
| Andolfi et al^26^ | 2016 | 79 | M | 1 | Yes | 3.3 | Yes | OR | No | No | 10 |
| Kutzner et al^27^ | 2017 | 61 | M | 1 | No | 2.2 | Yes | OR^††^ | No | No | 12 |
| Ryabov et al^28^ | 2017 | 55 | M | 1 | Yes | 4.3 | Yes | OR | No | No | 24 |
| Gao et al^29^ | 2017 | 60 | M | 1 | Yes | 2.6 | Yes | OR | No | No | 18 |
| Barisione^30^ | 2017 | 87 | M | 1 | Yes | NR | Yes | ED | Yes | RD | 12 |
| Terrington^31^ | 2018 | 67 | F | 1 | Yes | 2.6 | NR | OR | No | No | NR |
| Khan et al^32^ | 2018 | 60 | M | 1 | No | 3 | No^¶¶^ | OR^††^ | No | No | 12 |
| Radzhabova et al^38^ | 2019 | 74 | M | 2 | Yes | 2.3^†^ | NR | 1) OR, RT  2) OR | 1) No  2) No | 1) Local  2) No | 1) 45  2) 33 |
| Carretta et al^33^ | 2020 | 52 | M | 2 | Yes | 3.5 | Yes^¶¶^ | OR | No | No | 8 |
| Carretta et al^33^ | 2020 | 78 | M | 2 | NR | 4.0 | NR | ED | Yes | RD | 36 |
| Heuermann et al | 2020 | 66 | M | 2 | Yes | 5.6 | Yes | OR | No | No | 4 |

Supplemental Table 1: Demographic and tumor characteristics of 35 patients with tracheal chondrosarcoma. All studies included represent case reports (level 5 evidence). Abbreviations: NR, not reported; M, male; F, female; OR, open resection; ED, endoscopic debulking; ER, endoscopic resection; RT, radiation therapy; RD, residual disease

† Size reported as 6cm^3^. Converted to diameter approximating a spherical tumor

‡ Sets of reports describe same patient

§ Previously diagnosed with chondroma at the same site

¶ Data reported outside of original case report^7,12^

†† Patients undergoing tumor debulking or incomplete resection before definitive tracheal resection

‡‡ Medically unfit for definitive surgery

§§ Refused re-resection of residual disease

¶¶ Unpublished data included with author’s permission
